# Developing and testing the effectiveness of a novel online integrated treatment for problem gambling and tobacco smoking: a protocol for an open-label randomized controlled trial

**DOI:** 10.1186/s13063-020-04867-1

**Published:** 2020-11-19

**Authors:** Elena Bilevicius, Alanna Single, Chris Baumgartner, Van Bui, Tyler Kempe, Michael P. Schaub, Sherry H. Stewart, James MacKillop, David C. Hodgins, Jeffrey D. Wardell, Roisin O’Connor, Jennifer Read, Heather Hadjistavropoulos, Christopher Sundstrom, Matthew T. Keough

**Affiliations:** 1 Department of Psychology, University of Manitoba, 190 Dysart Rd, Winnipeg, MB R3T 2N2 Canada; 2 Swiss Research Institute for Public Health and Addiction, University of Zurich, Konradstrasse 32, CH-8031 Zurich, Switzerland; 3 Departments of Psychiatry and Psychology & Neuroscience, Dalhousie University, Abbie J Lane Building, 8th floor, 5909 Veteran’s Memorial Lane, Halifax, NS B3H 3A7 Canada; 4 Peter Boris Centre for Addictions Research McMaster University/St. Joseph’s Healthcare Hamilton, 1280 Main St West, Hamilton, ON L8S 4L8 Canada; 5 Department of Psychology, University of Calgary, 2500 University D NW, Calgary, AB T2N 1N4 Canada; 6 Department of Psychiatry, University of Toronto, 250 College Street, Toronto, ON M5T 1R8 Canada; 7 Department of Psychology, Concordia University, 7141 Sherbrooke W., Montreal, Canada; 8 Department of Psychology, University of Buffalo, 231 Park Hall, Buffalo, NY 14260-4110 USA; 9 Department of Psychology, University of Regina, 3737 Wascana Parkway, Regina, SK S4S 0A2 Canada; 10 Department of Psychology, York University, 4700 Keele St, North York, ON M3J 1P3 Canada

**Keywords:** Problem gambling, Tobacco smoking, Integrated treatment, Cognitive behavioural therapy, Motivational interviewing, Online, Self-help

## Abstract

**Background:**

Gambling and tobacco smoking are highly comorbid among North American adults. However, there is a paucity of treatment options that are integrated (i.e. targeting both gambling and tobacco smoking simultaneously), accessible, and evidence based.

**Methods:**

The aim of this two-arm open-label randomized controlled trial is to examine the effectiveness of an online, self-guided integrated treatment for problem gambling and tobacco smoking. A target sample of 214 participants will be recruited and be randomized into either an 8-week integrated or gambling only control condition. Both conditions will consist of seven online modules following cognitive behavioural therapy and motivational interviewing principles. Our three primary outcomes are (1) the number of days gambled, (2) money spent on gambling activities, and (3) time spent in gambling activities. Secondary outcomes include gambling disorder symptoms, cigarette use, and nicotine dependence symptoms. Assessments will be completed at baseline, at completion (i.e. 8 weeks from baseline), and at follow-up (i.e. 24 weeks from baseline). Generalized linear mixed modelling will be used to evaluate our primary and secondary outcomes. We expect that participants receiving online integrated treatment will show larger reductions in gambling relative to those receiving a control gambling only intervention. We further hypothesize that reductions in smoking will mediate these group differences.

**Discussion:**

The rates of problem gambling and tobacco smoking are high in North America; yet, the treatment options for both are limited, with no integrated treatments available. If supported, our pilot study will be a cost-effective and accessible way to improve treatments for co-occurring problem gambling and tobacco use.

**Trial registration:**

ClinicalTrials.gov NCT03614884. Registered on August 3, 2019

## Background

### Significance

Problem gambling [1, 2] and tobacco smoking [3, 4] are highly comorbid [5, 6] in North America. Indeed, studies show that tobacco dependence is the most common comorbid disorder among problem gamblers, with prevalence rates ranging from 41 to 60% [7–11]. According to the World Health Organization, tobacco use kills up to 50% of its users—translating into nearly six million deaths annually [12]. Tobacco use is also linked to several chronic health conditions, including cancers, respiratory problems, and cardiovascular diseases [12]. Given their high rates of smoking (relative to the general population) [7–9], problem gamblers are thus disproportionately affected by the increased morbidity and mortality from tobacco use. Moreover, research to date shows that co-occurring tobacco use compounds gambling-related harms. Problem gamblers who smoke have more severe gambling pathology [13], experience stronger gambling urges [14], are more likely to have other mental disorders [13], tend to bet larger sums of money and spend more time in gambling activities [15], and have greater financial problems [16]. Based on this, it has been suggested that daily smoking, a central trigger for gambling and related cravings, may undermine the treatment of problem gambling [5]. Accordingly, a priority needs to be placed on integrating the treatment of tobacco smoking into evidence-based interventions for problem gambling. In this study, we aim to design and test a novel online pilot study for comorbid problem gambling and tobacco use.

The proposed open-label pilot intervention will draw on strategies from cognitive behavioural therapy (CBT) and motivational interviewing (MI)—both evidence-based psychotherapies for problem gambling [17–19] and smoking [20, 21]. A main strength is that this pilot study will be integrated—meaning that it will use treatments for both problem gambling and tobacco smoking to target the functional relations between each behaviour within the same treatment. This is in contrast to limited traditional approaches, which include treating these problems either one at a time or simultaneously by two different professionals. The online platform also offers key advantages relative to in-person modalities. First, some provinces across Canada (e.g. Manitoba and Saskatchewan) and states in the USA have significant rural spread, meaning that communities are dispersed throughout the area with little access to major cities. This poses huge challenges for providing equal access to mental health care services for all citizens. In fact, statistics suggest that people living in remote communities struggle most with addictive behaviours and mental health issues but have limited access to treatment facilities [22]. Thus, we will be better able to reach these people with an online treatment. Second, many problem gamblers do not seek traditional forms of treatment due to stigma [23]. Problem gamblers may be more willing to try online interventions due to anonymity and reduced shame [24]. This integrated intervention has the potential to have positive impacts on the health of adult North Americans who struggle with problem gambling and tobacco use.

### Evidence for the association between problem gambling and tobacco smoking

Based on the epidemiological literature showing very high prevalence rates of tobacco smoking among problem gamblers, researchers have begun to examine the potential reasons for this association. While we still do not know the exact mechanisms underlying the problem gambling-tobacco use comorbidity, neurobiological studies suggest that both addictive behaviours are mediated by similar reward circuits in the brain [5]. Specifically, neurobiological work shows that drugs of abuse, including nicotine, increase transmission of dopamine in mesocorticolimbic regions [25, 26]. This effect is thought to underlie the reinforcing properties of substance use. Similarly, data suggest that gambling is also associated with increased activity in dopaminergic-rich areas of the mesocorticolimbic circuit. For example, in a double-blinded laboratory study [27], it was shown that administration of amphetamine (a potent dopamine agonist or releaser) increased motivation to gamble among individuals with gambling problems. Taken together, tobacco use and problem gambling appear to act on similar neural pathways that underlie addictive, reward-driven behaviours.

Functionally, research shows that nicotine may enhance or augment the reinforcing value of other addictive behaviours [5, 28]. Nicotine has been shown to lead to increased self-administration of alcohol in male smokers [29], increased cravings in cocaine-dependent smokers [30], and increased self-administration of methadone in opioid-dependent smokers [31]. Compared to alcohol and other drugs, very little neurobiological work has been done to show direct functional effects of nicotine on the reinforcing qualities of gambling. Indirect evidence for nicotine’s effects on gambling behaviour, however, comes from studies examining the impact of monetary reward on dopaminergic transmission in the brain. Overall, this work shows that when individuals receive uncertain or variable monetary rewards (i.e. those that cannot be predicted by any regularity), they show increased dopaminergic activity in the very same regions associated with tobacco use [32, 33]. In comparison, when monetary rewards are certain or predictable, there is no increased dopaminergic transmission in these brain regions. Given that uncertain or variable reinforcement is a hallmark of gambling, one could predict that co-occurring tobacco use could promote or reinforce gambling and gambling-seeking behaviours over time (via nicotine’s effects on dopaminergic brain regions in the mesocorticolimbic pathway).

Complementing neurobiological studies, behavioural research demonstrates that nicotine may alter reward-related cognitive processes that increase risk for problem gambling [5]. To illustrate, nicotine may enhance the salience of short-term rewards from gambling, while detracting focus from gambling’s longer-term negative outcomes. This notion is supported by work showing that heavy smokers engage in risky decision-making on the Iowa Gambling Task, with response patterns showing a preference for short-term gains at the expense of long-term losses [34]. Heavy smokers also show steeper discounting of future rewards relative to non-smokers [35], suggesting that nicotine may reinforce impulsive behaviour—like gambling—where the goal is the immediate reward. It is possible that tobacco smoking (via nicotine’s effects on learning and reward systems in the brain) strengthens problem gambling, making this behaviour difficult to extinguish especially when repeatedly paired with cigarette use. This may account for the increased clinical severity in heavy smoking (relative to non-smoking) problem gamblers. Moreover, some studies demonstrate that nicotine may enhance cognitive processes, like attention and executive control [36]. These momentary effects may be highly desirable to problem gamblers, as they may experience a greater ability to focus and shift attention during gambling episodes (after smoking).

Finally, the literature on cross-cue reactivity shows that tobacco use and problem gambling may become powerful reciprocal triggers for each behaviour [37]. That is, through repeated co-occurrence, stimuli associated with smoking are believed to become conditioned stimuli for gambling and vice versa. For example, over time, smoking cues can come to elicit strong urges to gamble, and conversely, gambling cues can come to promote cravings for tobacco use. While this is a relatively understudied research area, recent data show that gamblers who smoke had greater cross-cue reactivity (compared to gambling and smoking only control groups) [37]. Results suggested that smoking gamblers had increased physiological arousal and greater subjective desire to smoke, irrespective of whether cues were smoking- or gambling-related [37]. Accordingly, if tobacco use potentiates gambling—and vice versa—then it would be very challenging for a person to reduce either behaviour in isolation.

### Existing evidence-based treatments for problem gambling

Existing evidence-based treatment protocols for problem gambling generally combine strategies from two main psychological intervention frameworks: CBT and MI [17, 38]. CBT is a structured and goal-oriented treatment, where individuals acquire skills to reduce problem gambling through modifying thoughts and behaviours in response to internal (e.g. negative emotions) and external (e.g. gambling cues) triggers. During CBT, individuals with gambling problems strengthen coping skills by completing various exercises both in-session and at home between sessions. Complementing CBT, MI strategies are used in gambling treatment to elicit and motivate positive change. MI is a patient-centred and collaborative approach, where the goal is to help patients resolve ambivalence about change and get them to move in a direction that is consistent with personal values. MI is typically a prelude to CBT, but also a style that a therapist can return to if barriers are encountered during CBT. The weight of the evidence demonstrates that the combination of CBT and MI has synergistic beneficial effects on gambling and smoking behaviours during treatment [17–19, 21, 38]. By increasing motivation for change using MI, individuals with problem gambling may be more willing to engage in the effortful activities of CBT (e.g. homework), which in turn, are essential for building better coping skills. Integrated MI may also help to clarify a problem gambler’s core values in CBT by creating a discrepancy between current and desired behaviour. Thus, from a theoretical perspective, CBT and MI naturally complement each other in the treatment of problem gambling. Supporting this, a recent meta-analysis showed that CBT/MI treatments reduce problem gambling symptoms with medium effect sizes [17], and online CBT treatments reduce problem gambling amount, frequency, and urges [39].

Despite CBT/MI’s effectiveness for reducing gambling, there are notable problems with existing approaches. First and foremost, while CBT/MI approaches have been shown to be helpful, effect sizes on short- and long-term gambling outcomes are modest [17]. This suggests that there is a great deal of room to improve interventions for gambling. Second, dropout rates in problem gambling treatment studies are substantial. Specifically, it has been estimated that 14 to 50% of individuals with problem gambling drop out of active treatment, with the average being about 30% across studies [40, 41]. This suggests that a large portion of treatment-seeking individuals with problem gambling do not complete this efficacious psychological intervention. Third, individuals with problem gambling show marked problems with treatment adherence, as evidenced by poor homework completion and low session attendance [42]. Additionally, comprehensive self-guided treatments are shown to be as effective as face-to-face treatments for problem gambling [43]. Adherence may be especially poor among the 50 to 70% [44] of problem gamblers with co-occurring substance use problems. In turn, poor treatment adherence predicts poor responses to intervention [42, 45]. Finally, similar to people with tobacco use disorders [46], relapse rates among treated problem gamblers remain very high [47]. As a whole, these issues suggest that we need to find effective ways to augment CBT/MI to improve clinical outcomes for problem gambling.

Based on the neurobiological and behavioural literature discussed above, it is highly possible that comorbid tobacco use is a factor that helps to maintain and reinforce problem gambling behaviours—even after treatment engagement. Despite their best efforts in treatment, individuals with problem gambling who smoke (versus those who do not smoke) may have marked difficulty controlling gambling urges, forming new non-gambling-related associations, and shifting focus to adaptive future (relative to often maladaptive and immediate) goals in therapy [5, 35, 36]. These difficulties may contribute to commonly observed poor treatment engagement and completion and the modest success of problem gambling treatment among individuals with problem gambling. Unaddressed daily tobacco use may also be a critical factor in high rates of relapse among treated problem gamblers. Very few studies have explored smoking status and its relationship to gambling-related treatment outcomes [48]. As noted earlier, tobacco-related cues are powerful conditioned stimuli that elicit strong cravings among problem gamblers [37]. It follows that even after treatment, individuals with problem gambling who continue to smoke will have to fight against strong urges resulting from their increased cross-cue sensitivity. Thus, a key augmentation to CBT/MI treatments for problem gambling would be to include content to address co-occurring tobacco use.

### Integrated treatment

Research on integrated addiction treatment is relatively new. This is surprising, given that it is common for individuals to present with more than one addictive behaviour [49]. Polysubstance use is associated with greater clinical severity and poorer treatment outcomes [50]. Further, poor treatment outcomes have also been observed for gamblers with substance abuse treatment history compared to gamblers without substance abuse treatment history [51]. Traditional methods for treating co-occurring addiction/mental disorders are sequential and parallel intervention [49]. During a sequential approach, clinicians treat the addiction/disorder viewed as “primary” first, followed by the treatment of the comorbid condition. For example, a person with co-occurring alcohol misuse and problem gambling would likely not be able to work on reducing gambling until they achieve some notable period of abstinence from drinking. Thus, in the sequential model, treatment is provided for one disorder at a time—with the more acute disorder (e.g. alcohol misuse) taking first priority. The sequential model of intervention has been (and still is) the most widely used approach to treating disorder comorbidities. In contrast, the parallel model involves treating co-occurring problems separately by two distinct professionals and/or clinical teams, each with expertise in one of the two problems [52]. An example of this approach would be a person seeing a family doctor for management of smoking, while working with a psychologist to reduce gambling. Therefore, in the parallel model, an individual receives support for both issues simultaneously, but from distinct professionals.

Although still widely used, sequential and parallel approaches are limited as intervention models for comorbid addictive behaviours [52]. A sequential approach may be necessary in crisis situations, such as when a person needs hospitalization for alcohol-related seizures. However, in the absence of an emergency warranting the immediate stabilization of one disorder over the other, sequential treatment may impede the treatment of both addictive behaviours [52]. Sequential treatment does not consider the interconnectedness of addictive behaviours. To illustrate, a person would likely find it very challenging to reduce gambling if their smoking (a main trigger for gambling) remains untouched in treatment. In turn, this person’s smoking (perhaps as a coping mechanism) would likely be worsened by repeated failed attempts to control gambling. In such a scenario, it would be difficult for this person to make major improvements on either problem. Moreover, in a parallel treatment model, there is often little communication between the professionals independently treating each problem [52]. This is problematic because professionals often have different case conceptualizations and treatment recommendations. For example, a physician may emphasize the usefulness of medication over psychotherapy, whereas the reverse may be true for a psychologist. Hence, it is very common for a person to get conflicting advice and feedback in a parallel treatment approach [52]. Furthermore, it is up to the patient to “integrate” distinct treatment approaches, which is likely difficult due to high rates of cognitive impairment among those with problem gambling [53]. Finally, another potential problem for the client is the demands of attending two separate treatments (i.e. time, money). The limitations of a parallel approach may lead to adverse patient outcomes, such as frustration, continued mental health challenges, and in the most extreme case, discontinuation of treatment. Overall, attesting to these limitations, the literature shows that sequential and parallel approaches result in poor treatment outcomes in those struggling with addictive behaviours [52].

The main advantage to an integrated treatment framework is that it recognizes common etiological mechanisms underlying co-occurring addictive behaviours. Hence, from a common mechanism or “transdisease” approach [54], one can design a treatment that helps individuals achieve notable improvements on more than one addictive disorder at a time. We posit that combined CBT/MI represents a general framework to target co-occurring problem gambling and tobacco use. CBT/MI therapies have been shown to be effective for smoking cessation across many studies [55], and these approaches are very similar in content to those used for problem gambling. Overall, CBT/MI therapies help people acquire coping skills to deal with addictive behaviours broadly, including building motivation, improving understanding of triggers (i.e. learning how smoking is a trigger for gambling), avoiding high-risk situations, developing balanced ways of thinking, and creating well-informed relapse prevention plans. Numerous articles support the efficacy of these CBT/MI strategies for the treatment of problem gambling, tobacco use, and addictive behaviours more broadly [17, 55]. In the gambling literature, approaches aimed at targeting both problem gambling and alcohol use [56] or mental health difficulties [57] were shown to have no significant difference in outcomes between the separate and integrated treatment interventions; however, a limitation to both trials relied on a more sequential approach to treatment and lacked a stronger integrated component. Given CBT/MI’s emphasis on general coping skill development, we posit that it is an ideal framework for an integrated intervention for co-occurring problem gambling and tobacco use.

### The current study

This open-label pilot study will address a notable gap in the literature on problem gambling treatment. It has been known for a long time that a high proportion of gamblers smoke cigarettes [7, 8] and that daily smoking compounds gambling severity [13–16]. However, very little work has been done to systematically address the problem of smoking in treatment for problem gambling. In fact, to our knowledge, no existing treatment protocols have integrated content to help problem gamblers reduce smoking. Using a randomized controlled trial (RCT), we will be the first to design, implement, and test a novel online integrated treatment for problem gambling and comorbid smoking. Relative to traditional face-to-face approaches, there are distinct advantages to an online delivery of integrated treatment in North American. First, online interventions would be able to reach adults from rural and Northern communities across North America. We know from health statistics that the highest rates of addictive behaviours and addiction-related deaths exist in these communities [58, 59]. Second, adults with gambling issues may be more willing to engage in a self-help online intervention (relative to in person). This is because an online modality may be associated with reduced shame and stigma—which are known, persistent barriers to seeking treatment among problem gamblers [24]. Finally, online interventions could significantly reduce the burden on mental health care systems in North America. More people with gambling problems would be helped for much less cost relative to hospital treatments. Data show that cost-effective, online psychosocial interventions reduce problem gambling [60] and tobacco use [61], separately. Therefore, the literature supports the online modality as a means to deliver our proposed integrated pilot study.

### Objectives

Informed by the literature, our primary aim will be to examine if integrating treatment of comorbid tobacco smoking improves gambling outcomes among North Americans with problem gambling relative to a focus on problem gambling alone. Our second aim will be to test if reduced smoking explains (or mediates) the beneficial effects of the integrated treatment on gambling. We expect that participants receiving online integrated treatment will show larger reductions in gambling relative to those receiving a control gambling only intervention. We further hypothesize that reductions in smoking will mediate these group differences.

## Methods/design

### Design

**Fig. 1:**
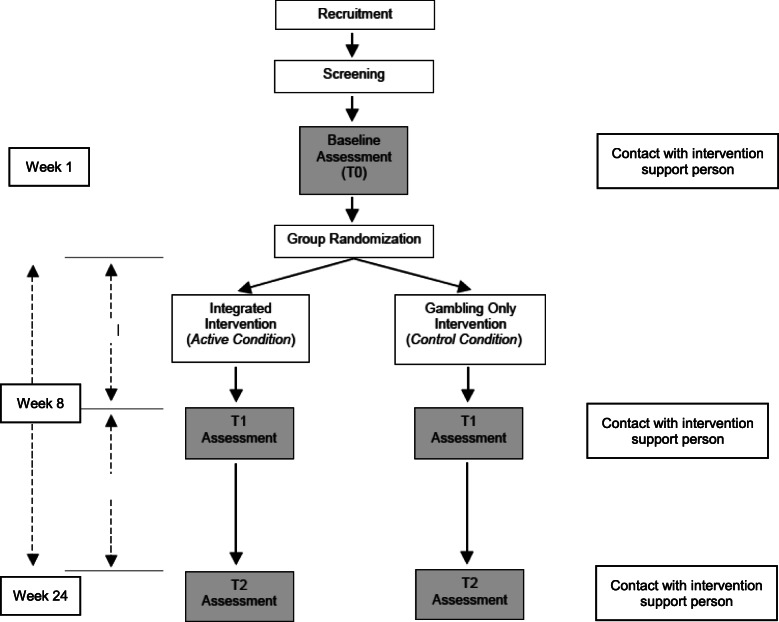
Schematic of the randomized controlled trial methodology

**Fig. 2:**
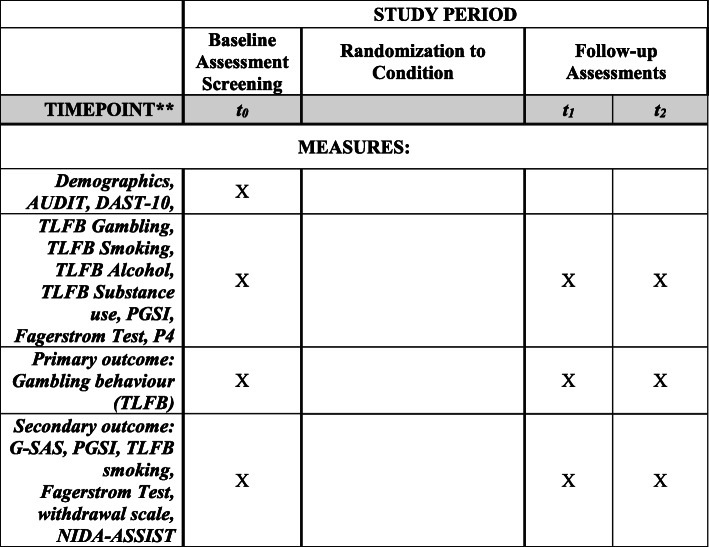
Schedule of enrolment, interventions, and assessments. *t*_0_, baseline (at outset of intervention); *t*_1_, 8 weeks (at completion of the intervention); *t*_2_, 24 weeks (at 4 months following completion of the intervention); AUDIT, Alcohol Use Disorder Identification Test; DAST-10, Drug Abuse Screening Test-10; TLFB, Timeline Followback; PGSI, Problem Gambling Severity Index; Fagerstrom test, Fagerstrom test of nicotine dependence; G-SAS, Gambling Symptom Assessment Scale; P4, P4 suicide screener; withdrawal scale, Hughes-Hatsukami withdrawal scale

This open-label pilot project will consist of a two-arm RCT (Clinicaltrials.gov ID NCT03614884; see Figs. 1 and 2 for an overview). Dr. Keough and his research team will collect the data in Canada and the USA. Participants will be randomly assigned into one of two conditions. In the experimental condition, participants will receive an 8-week online integrated intervention for problem gambling and smoking. In the control condition, participants will receive a similar 8-week online intervention for problem gambling without treatment content to address smoking. Online assessments will occur before randomization (T0; baseline), at 8 weeks since baseline (T1; treatment end), and at 24 weeks since baseline (T2; follow-up; see Table 1). The timeline of these assessments, including the longer-term follow-up, is consistent with previous RCTs examining technology-based interventions for problem gambling and those for smoking cessation [62, 63]. Participants will be compensated based on the following schedule: $20 per assessment, a bonus of $20 for completing all three assessments, and $20 for completing at least five of the treatment modules (max compensation per participant = $100). For ethical reasons, participants will be compensated in the form of gift cards to Amazon.ca. This RCT is registered with ClinicalTrials.gov for transparency and to avoid publication bias. Prior to the initiation of the study, we obtained ethical approval from the Research Ethics Board (REB) at the University of Manitoba (#P2018:088 HS22037) and York University (ES2020-006).

**Table 1:** Schedule of assessments for measures

Self-report measures	Baseline (T0)	8 weeks (T1)	24 weeks (T2)
1. Demographics (e.g. age, gender, treatment history, psychiatric/medical history)	X		
2. Alcohol Use Disorders Identification Test (AUDIT)	X		
3. Drug Abuse Screening Test-10 (DAST-10)	X		
4. Timeline Followback (TLFB) for gambling, smoking, alcohol, and substance use	X	X	X
5. Gambling Symptom Assessment Scale (G-SAS)	X	X	X
6. Problem Gambling Severity Index (PGSI)	X	X	X
7. Fagerstrom test for nicotine dependence	X	X	X
8. P4 suicide screener	X	X	X
9. Withdrawal scale	X	X	X
10. NIDA-ASSIST	X	X	X

### Recruitment

A target sample size of *N* = 300 will be recruited. Dr. Keough’s research team will oversee online data recruitment and collection. A variety of methods will be used to recruit potential participants. Dr. Keough will recruit from cities across Canada and the USA using online ads (e.g. Google Ads, Craigslist, Kijiji, and local news websites), local avenues (i.e. newspapers), and governmental organizations (i.e. Manitoba Liquor & Lotteries). Given the high rates of problem gambling and tobacco smoking in Canada and the USA [7–9, 12], it should be feasible to recruit the required sample size.

Another very important issue will be to recruit a sufficient number of women to be able to examine gender differences in treatment effects. Based on the literature, about one-third of individuals with problem gambling from the community will be women [64]. We will make every effort to ensure that at least one-third of our final sample is comprised of women, although we will aim for a higher percentage during the recruitment process. Inclusive recruitment will ensure that both genders equally contribute to and benefit from the intervention. This is very important, since data show that women are drastically underrepresented in gambling treatment settings. To illustrate, only 2–7% of those who attend Gambler’s Anonymous are women [65]. We aim to greatly improve these numbers for our trial.

### Inclusion criteria

Our main target demographic will be adults with moderate levels of gambling problems who identify as daily smokers. Inclusion criteria will include (1) ages 19+, (2) problem gambling status based on reporting a score of > 3 on the Problem Gambling Severity Index (PGSI), (3) reporting current daily smoking, (4) fluency in English, and (5) have weekly Internet access.

### Exclusion criteria

Exclusion criteria will include (1) self-reported current engagement in other psychosocial treatments for problem gambling and/or smoking, (2) elevated suicidality (scoring greater than “minimal risk” on the P4 suicide screener) [66], (3) past-90-day psychosis or mania, and (4) presence of a severe substance use disorder (SUD). SUD severity will be determined based on established cut-offs on widely used and validated screening measures for alcohol and other drug use. For alcohol, individuals with a score of > 20 on the Alcohol Use Disorder Identification Test (AUDIT) [67] will be excluded from the study. For other substance use, individuals with a score of > 5 on the Drug Abuse Screening Test-10 (DAST-10) [68] will be excluded. The rationale for excluding those with severe SUDs is based on the large literature showing that these individuals need intensive residential treatments (with corresponding medical supervision) [69] and hence would not benefit from self-guided online interventions, such as the one to be provided in the current study. We will provide a list of local addiction and financial resources for those with severe AUD and SUD symptoms.

### Sample-size justification and statistical plan

A meta-analysis showed that CBT/MI (relative to control) treatments have medium effects on gambling [17]. However, we will be comparing two active treatments, which may result in smaller effect sizes. Accordingly, we used G*Power to calculate the sample size needed to detect a small effect size for our primary outcome. We used a 2 (between subjects; treatment condition) by 3 (within subjects; time) mixed design. Assuming a power rate of .80, an *α* of .05, and a correlation of .50 between repeated measures, the required sample size to detect a small effect size is *N* = 164. Given an average attrition rate of 30% in gambling treatment studies [17], our final adjusted sample size is *N* = 214. Generalized mixed modelling will be used to evaluate the main hypothesis that integrated online treatment will result in the larger reductions in gambling than the gambling only treatment. Primary and secondary outcomes will be tested sequentially. We will include all randomized participants in analyses (i.e. intent-to-treat). Missing data will be accounted for using full information maximum likelihood estimation. We will include some covariates in our models if needed. Relevant covariates may include history of psychological treatment, age, gender, and baseline severity of gaming and smoking behaviours. Of particular relevance, we expect variability in adherence to NRT; therefore, we will explore whether NRT use (e.g., # of days patch was used during treatment) affects primary/secondary treatment outcomes.

#### Primary outcomes

##### Gambling behaviour

There will be three primary outcomes to capture intervention-related change in gambling behaviour. Specifically, participants will be asked about their past-30-day gambling frequency (# of days gambled), money spent on gambling activities (in dollars), and time spent engaged in gambling activities (# of minutes). These primary indices were selected because they are common outcomes in RCT studies examining CBT/MI approaches to the treatment of problem gambling [17, 70]. The Timeline Followback (TLFB) procedure will be used to collect data for the primary outcomes [71]. Anchoring gambling to specific calendar dates helps to improve the accuracy of self-reported involvement in these activities. The TLFB procedure has also been shown to produce relatively accurate estimates of addictive behaviour involvement when participants are ensured anonymity [72]. Furthermore, recent data shows that a self-report web-delivered version of the TLFB (like the one to be used in this study) produces reliable and valid estimates of addictive behaviours [73].

#### Secondary outcomes

##### Gambling Symptom Assessment Scale (G-SAS)

The G-SAS is a 12-item self-report questionnaire that was designed specifically to capture change in gambling symptoms following treatment [74]. The G-SAS has a unidimensional structure, and psychometric work shows that G-SAS total scores have good reliability and good convergent validity with clinician-rated measures of gambling symptom change during treatment, such as the Yale-Brown Obsessive Compulsive Scale modified for Pathological Gambling [74]. The main advantage of using the G-SAS as a secondary measure of gambling symptom change is that it includes several questions about gambling urges. While urges are expected to decrease with intervention, existing widely used measures (e.g. Problem Gambling Severity Index [PGSI]) do not include items to assess urge change during treatment. The G-SAS also includes items to assess changes in gambling harms during treatment (e.g. reductions in emotional distress). In the present study, the total G-SAS score will serve as a secondary measure of gambling change after intervention.

##### Problem Gambling Severity Index (PGSI)

The PGSI is the most widely used self-report measure of gambling harms in the literature [75]. The PGSI contains nine items that assess a broad array of problems experienced by individuals who engage in problem gambling (e.g. guilt, financial problems etc.). Previous work supports the PGSI’s good reliability and validity [75]. The total PGSI score will be used in this study to capture changes in gambling harms following the intervention.

##### Cigarette use

The TLFB procedure will also be used to collect information about past-30-day cigarette use. A sum score will be used to reflect total smoking behaviour at each assessment. This secondary outcome will be used to capture intervention-related change in smoking behaviour. As noted above for gambling, recent data show that a self-report web-delivered TLFB provides valid estimates of cigarette use [73]. In addition to collecting information on cigarette use, we will also use the TLFB procedure to code for episodes of co-occurring gambling and smoking. That is, participants will be asked to denote the days in which they smoked while gambling (e.g. leaving a bar with VLTs momentarily to smoke, then resume playing). This variable may be used for secondary analyses, depending on the variability in the data.

##### Nicotine dependence symptoms

The Fagerstrom Test of Nicotine Dependence is a six-item self-report measure that captures the severity of nicotine dependence symptoms [76]. The Fagerstrom total score will be calculated at each time point and will be used to assess intervention-related changes in nicotine dependence symptoms. Previous work shows that the Fagerstrom has good reliability and validity [76] and is sensitive to treatment-induced change [77].

##### Withdrawal scale

The Hughes-Hatsukami Withdrawal Scale [78] is a 13-item self-report measure that assesses symptoms of tobacco withdrawal. Previous work shows that the Hughes-Hatsukami Withdrawal Scale has good reliability and validity [78].

##### NIDA-ASSIST

The National Institute on Drug Abuse—Alcohol, Smoking and Substance Involvement Screening Test [NIDA-ASSIST] [79] is a 10-item scale that assesses the level of risk associated with different substance involvement. A total score will be computed for drug use.

##### P4 suicide screener

The P4 suicide screener [66] is 4-item self-report measure that evaluates an individual’s level of suicide risk. It assesses past suicide attempts, suicide plan, probability of completion, and preventative factors. Individuals who endorse one or more of the aforementioned criteria are deemed to be greater than minimal risk and will be excluded from the intervention. The P4 suicide screener has good reliability and validity [66].

### Informed consent and randomization

Participants will first read the study rationale on the intervention website and then will be told the following during informed consent: (1) the inclusion/exclusion criteria, (2) the potential risks/benefits, (3) the safety arrangements during and after the study, (4) the programme is self-guided, (5) the circumstances under which they should contact a medical professional from an emergency list that will be accessible at all times via the menu item “Help Me” on the intervention website, and (6) participation is voluntary. Participants endorsing significant suicide ideation and/or plans will be told to visit a hospital for support. For ethical reasons, individuals with clinically elevated SUD/AUD symptoms (i.e. those who score above the cut-offs on our screening tools) will be given access to the integrated intervention and will be given the contact information for mental health professionals in their area. However, they will be excluded from the study. Following informed consent, participants will register on the intervention website and complete baseline assessments to determine eligibility using inclusion/exclusion criteria. After completion of the baseline measures, a computer programme will be used to randomize eligible participants to the treatment conditions. Once participants are randomized, an intervention support person will be aware to their respective conditions (i.e. unblinded). Additionally, participants will also be aware of their assigned condition. It would be difficult to achieve blindness as both participants and intervention support personnel will be aware of the condition through the delivery of NRT.

Prior to starting the pilot study, an intervention support person will contact all participants via phone or email to confirm eligibility and willingness to participate. Contact information (i.e. phone number and chosen email) will be collected via the intervention website and is stored separately from participant data. It is important to note that the intervention support person does not have any formal training in clinical psychological treatment to ensure that participants were not inadvertently receiving psychotherapy. The role of the intervention support person is to strictly answer participant-initiated questions about the website and pilot study.

### Integrated treatment (experimental condition)

**Fig. 3:**
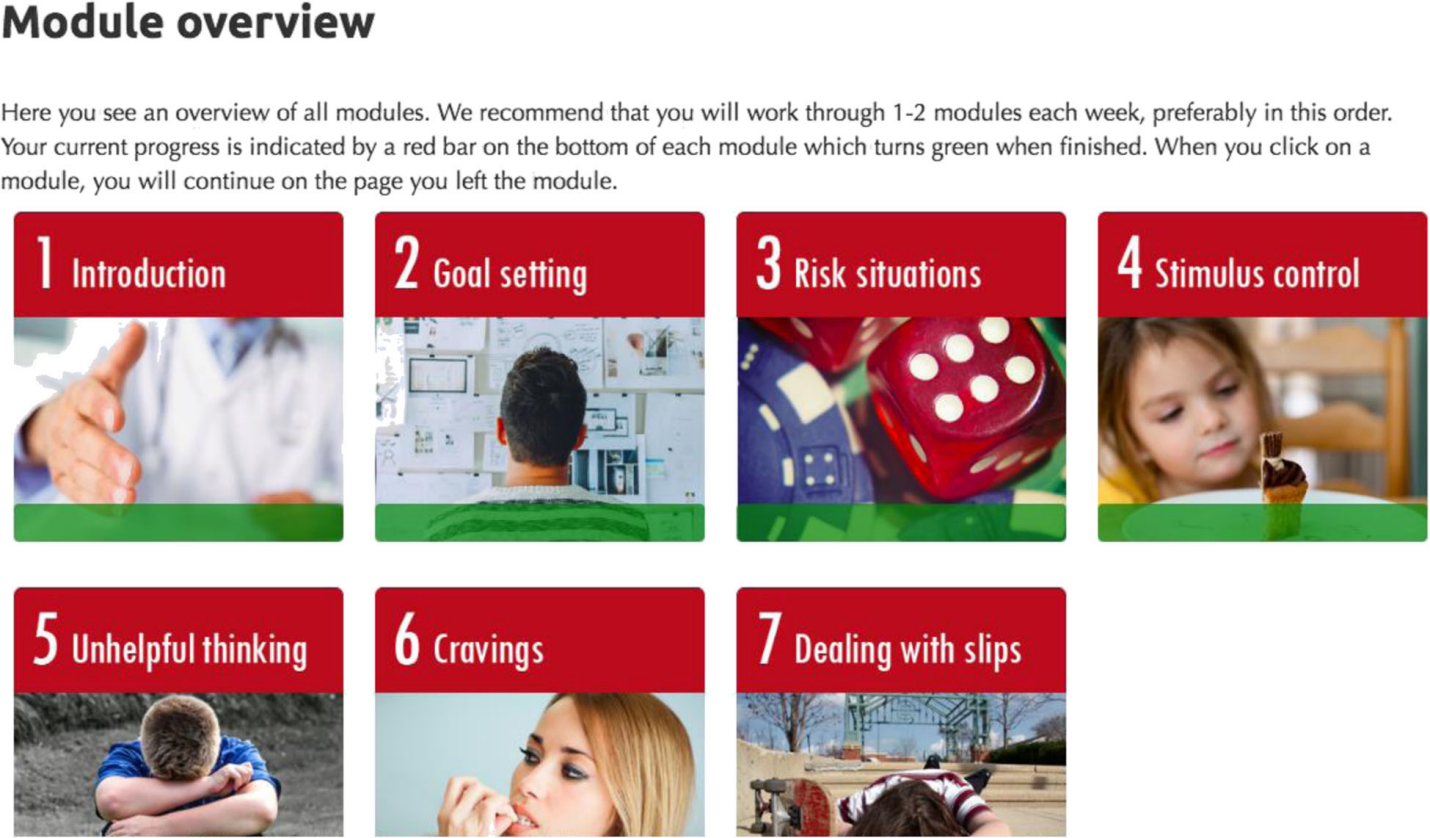
Main menu of intervention modules

Participants in this condition will have access to seven treatment modules over 8 weeks (see Fig. 3 for the overview of the module interface). The specific gambling content will be adapted from a self-help intervention developed by Dr. Hodgins (a co-investigator on the proposed project; see Table 2 for module content for integrated intervention). This intervention has been validated in several trials [38, 70] and has been successfully adapted for online delivery [55, 80]. Smoking content will be adapted from existing evidence-based CBT/MI protocols, including the most widely used best practice guidelines from both Abrams et al. and Fiore and colleagues [55, 81]. One standard care guideline for smoking cessation interventions is the combined use of nicotine replacement therapy (NRT) and psychosocial support (CBT/MI) [55, 81]. Accordingly, in the first module, participants in the integrated treatment arm will be provided with an extensive fact sheet on NRT use—including the strong evidence supporting its use in conjunction with psychosocial treatment for smoking cessation. Participants will be (1) encouraged to use NRT patches for the 8 weeks of active treatment, (2) advised that NRT patches are available over the counter at any local pharmacy, (3) provided with NRT patches for the duration of treatment, and (4) advised to consult with their family physician should they have any medical questions related to NRT patches. As noted in best practice guidelines [81], the dosage schedule of NRT patches will be the following: 24 mg for 4 weeks then 14 mg for 2 weeks and finally 7 mg for the remaining 2 weeks. NRT will be mailed out to each participant at the outset of their registration to ensure that they have it for the duration of the pilot study. NRT usage will be tracked weekly during the active intervention and also at each follow-up assessment.

**Table 2:** Module content for integrated intervention

Module number and title	Module content
M1: Introduction	• Introduction to the intervention and website
	• Psychoeducation about the links between gambling and cigarette smoking, including a rationale for working on both in treatment together
	• Motivational enhancement (i.e. identifying reasons for change, and pros and cons of gambling/not gambling, and smoking/not smoking)
	• Psychoeducational fact sheet on nicotine replacement therapy (NRT), including a strong recommendation for using NRT during the intervention
	• Self-monitoring gambling and smoking behaviours, including frequency of co-occurrence
M2: Goal setting	• Determine personal goals related to gambling and smoking (i.e. abstinence versus harm reduction)
	• Develop SMART (specific, measurable, achievable, realistic, and timely) goals for gambling and smoking reduction
M3: Risk situations	• Resisting gambling in specific situations (e.g. situations involving negative emotions)
	• Developing personal strategies to reduce/abstain from harmful gambling and to control access to money
	• Introduce need for pleasurable activity scheduling (i.e. behavioural activation)
	• SMART goal setting for pleasurable, low-risk, and healthy activities (e.g. activities that are incompatible with gambling and smoking)
M4: Identifying triggers	• Strategies for avoiding triggering situations related to smoking
	• Identify strategies for refusing smoking and gambling
M5: Unhelpful thinking	• Review common thinking errors related to gambling (or “gambling traps”) and smoking
	• Psychoeducation about games of chance
	• Identify how misperceptions about gambling odds impact one’s inner dialogue or thoughts
	• Psychoeducation about thinking traps related to smoking
M6: Cravings	• Psychoeducation about craving
	• Introduce self-monitoring of craving
	• New ways to effectively cope with cravings (e.g. distraction, urge surfing, and recalling the negative outcomes of gambling)
	• Identifying the similarities and differences between gambling and smoking cravings
M7: Dealing with slips	• Define a “slip” versus a full-blown relapse
	• Introduce ways to cope with slip in meeting gambling and smoking goals
	• Introduce relapse prevention planning
	• Create personalized relapse prevention plan
	• Identify three coping strategies for preventing gambling and smoking

The psychosocial integrated treatment content is fully summarized in Table 2. As noted earlier, all components of this pilot study come from well-established CBT/MI protocols for problem gambling and smoking cessation [17, 55]. Integrated content will allow participants to understand the links between gambling and smoking; identify and set goals related to gambling and smoking, as well as make goals for increasing involvement in pleasurable and positive activities; learn to identify and plan for “high risk” situations related to smoking and gambling; develop strategies to cope with gambling and smoking urges; learn to challenge and/or alter thoughts that promote gambling and smoking; and learn how to prevent relapse of each behaviour. Participants will have immediate access to all modules, and it will be recommended that they work through the modules sequentially. However, any order is possible. An adult might, for example, jump ahead to a module on craving, if they are having strong urges to smoke in a given week. Participants will be encouraged to complete the modules as many times as needed, and their progress will be visible on a digital progress bar. If participants leave a module and revisit it later, they will restart where they left off. Participants will be asked to track both gambling and smoking behaviours each week. On the “dashboard” intervention page, participants will be able to see a graph depicting their individualized treatment progress for their gambling and smoking behaviour. The website will automatically adapt for use on smartphones and tablets.

### Gambling only treatment (control condition)

The control group will receive a similar 8-week online intervention for gambling only. The gambling content will be the same as in the experimental condition, but there will be no treatment content about smoking and no integrated content. The complete module content for the gambling only (control) arm is summarized in Table 3.

**Table 3:** Module content for gambling only (control) intervention

Module number and title	Module content
M1: Introduction	• Introduction to the intervention and website
	• Motivational enhancement (i.e. identifying reasons for change, and pros and cons of gambling and not gambling)
	• Self-monitoring gambling behaviours
M2: Goal setting	• Determine personal goals related to gambling (i.e. abstinence versus harm reduction)
	• Develop SMART (specific, measurable, achievable, realistic, and timely) goals for gambling reduction
M3: Risk situations	• Resisting gambling in specific situations (e.g. situations involving negative emotions)
	• Developing personal strategies to reduce/abstain from harmful gambling and to control access to money
	• Introduce need for pleasurable activity scheduling (i.e. behavioural activation)
	• SMART goal setting for pleasurable, low-risk, and healthy activities (e.g. activities that are incompatible with gambling)
M4: Cravings	• Psychoeducation about craving
	• Introduce self-monitoring of craving
	• New ways to effectively cope with cravings (e.g. distraction, urge surfing, and recalling the negative outcomes of gambling)
M5: Unhelpful thinking	• Review common thinking errors related to gambling (or “gambling traps”)
	• Psychoeducation about games of chance
	• Identify how misperceptions about gambling odds impact one’s inner dialogue or thoughts
	• Introduce the thought record as a way to see associations between situations, automatic thoughts, behaviours, and gambling consequences
	• Help foster identification of “gambling traps” and strategies to challenge them
M6: Dealing with slips	• Define a “slip” versus a full-blown relapse
	• Introduce ways to cope with slip in gambling
	• Introduce relapse prevention planning
M7: Preserve your success	• Identify “early warning signs” for slip/relapse
	• Create personalized relapse prevention plan
	• Discuss ways to cope with relapse
	• Identify top five coping strategies for preventing gambling
	• How to know if more treatment is needed (with corresponding recommendations)

## Discussion

### Possible challenges and mitigation plan

#### Adherence to treatment

The literature shows that adherence to treatment tends to be suboptimal in addictive behaviour intervention studies, including both in-person and online modalities [82]. To address this potential challenge, the proposed intervention will have features aimed at improving adherence, including automatic reminders to complete modules and assessments. Inclusion of these strategies within this pilot study was based on literature showing that frequent automatic and specific feedback improves adherence to self-guided behavioural change interventions [83].

#### Recruitment

As noted in the methodology section, Dr. Keough and his research team will make every effort to recruit adults across North America. Our intent is to recruit using as many different recruitment strategies as possible, such as through Web-based advertisements (e.g. Google Ads, Kijiji ads, Craigslist), local avenues (e.g. posting recruitment flyers in public spaces), through social media (e.g. Twitter, Facebook), and using community and university organizations to promote the intervention. Recruitment posters will also be posted at popular gambling locations (e.g. restaurants and bars). This will help to increase recruitment in each province. However, it is possible that issues with suboptimal recruitment will occur. Recruitment rates will be monitored on an ongoing basis (e.g. % completing screening relative to % meeting criteria to participate).

#### Attrition

Intervention studies show substantial variability in attrition rates, depending on treatment orientation, clinical severity, and so forth [17]. Generally, dropout rates are high in populations with behavioural and substance use addictions—ranging from 21 to 80% [84]. We are aware of this possible influence on attrition. Regular contact will be provided via automated emails and, if a participant prefers, phone to provide personalized support that is not typical of online interventions. It has been shown that automated and personalized support in self-guided interventions can improve treatment adherence and reduce attrition [85]. This may reduce dropout. Finally, we will offer participants the possibility to receive a personalized feedback report at the end of the study. This report will contain information about their treatment progress. The combined use of the strategies above will help reduce the attrition rate.

### Methods: monitoring

A formal data monitoring committee (DMC) was not included in the present RCT due to budgetary reasons (i.e. there was no dedicated funding to compensate independent committee members). Instead, we opted to include co-investigators on this trial who have extensive experience in conducting online treatment RCTs. This study was also intended to be an open-label pilot intervention, and the plan is for subsequent larger trials to have a dedicated DMC. We do not intend to conduct any interim analyses nor have any stopping guidelines been established. Safety monitoring and reporting adverse events will be the responsibility of the trial principal investigator, Dr. Keough. At present, there are no plans to externally audit the trial.

### Ethical considerations

The intervention will be designed to adhere to the ethical principles of the Declaration of Helsinki. Ethics approval will be obtained from the REB at the University of Manitoba and York University before study initiation. Participant data will be kept confidential. To achieve this, we will be using arbitrary identifiers (e.g. numerical IDs) for participants. These identifiers will be used to link data over time, but the data set will not contain personal information. We will keep a master list of participant contact information in a separate password-protected master list. We are also aware of the increased risks associated with recruiting adults with clinically elevated gambling problems, considering the high rates of comorbid mental health problems with disordered gambling [86]. We would expect higher base rates of suicidality and self-harming behaviours in these individuals relative to those without these substance use and behavioural addiction concerns. Accordingly, we will have safeguards to minimize risk of harm. Adults who report significant suicide ideation and/or plans during screening will be given a recommendation to visit their local medical professional (GP or hospital) for support. They will also be given access to the integrated intervention, but their data will not be analysed. We will also be monitoring changes in suicidality at each assessment and will direct participants to emergency services if needed. Participants will also have full-time access to a list of mental health services listed on the intervention website, including community resources, public and private psychologists, hospitals, and helplines. Dr. Keough will be available to speak with participants if serious issues arise (i.e. increase in suicidal ideation) and will make sure a safe course of action is followed. The reporting of harms is the responsibility of Dr. Keough as outlined in the approved ethics documents. If a participant feels uncomfortable at any time during treatment, they are free to withdraw and cease their involvement in the trial. For ethical reasons, people with clinically elevated AUD/SUD symptoms will be provided with access to the integrated treatment instead of being denied access should they require the intervention as a resource for their problem gambling and/or smoking behaviours. However, their data will not be included in the study. Finally, participants in the gambling only control condition will be given access to the active integrated treatment after the final assessment (6 months) should they wish. They will receive all elements of the integrated arm, including NRT.

### Implications of the proposed pilot study

High rates of problem gambling and tobacco use in North America demonstrate a clear need for more mental health services in the country around this crucial issue. Some provinces and states also have significant rural spread, meaning that several communities are dispersed throughout the province with little access to major city centres. This poses a huge challenge for providing equal access to mental health care services for all North Americans. In fact, statistics suggest that people living in remote communities are at a significant disadvantage [7, 8]. They seem to be struggling most with addiction and related problems, but have limited access to treatment facilities. Thus, the proposed study has the potential to substantially improve the health and well-being of adults living across the continent. Furthermore, if supported, the proposed intervention would be a cost-effective method (relative to traditional in-person treatments) of improving gambling addiction care delivery to North Americans. Online interventions have the potential to save the government thousands of dollars via the reduced burden on the health care system. We plan to disseminate the results of the pilot study widely, which will include giving talks at Manitoba Blue Cross/AFM locations across the province, at major hospitals and universities in Manitoba, and at public institutions (e.g. libraries). We will publish results in open-access journals, so that the public and policymakers in North America can learn about (and use) the intervention.

### Trial status

To ensure transparency, the current study has been registered on the clinicaltrials.gov website (Clinicaltrials.gov ID NCT03614884). Any protocol modifications will be updated on clinicaltrials.gov and on the consent form located on the online intervention website.

Protocol version number: NCT03614884.

Date trial was registered: August 3, 2018.

Date recruitment began: May 2, 2019.

Last update posted: September 25, 2019.

Estimated date when recruitment will be completed: December 31, 2020.

## Supplementary Information

**Additional file 1.** Informed Consent Form

## Data Availability

The datasets generated and/or analysed during the current study are not publicly available due to ongoing data collection, but are available from the corresponding author on reasonable request. Results will be published in open-access journals.

## Abbreviations

RCT: Randomized controlled trial
CBT: Cognitive behavioural therapy
MI: Motivational interviewing
REB: Research ethics board
MGRP: Manitoba Gambling Research Program
AUDIT: Alcohol Use Disorder Identification Test
DAST-10: Drug Abuse Screening Test-10
AUD: Alcohol use disorder
SUD: Substance use disorder
G-SAS: Gambling Symptom Assessment Scale
TLFB: Timeline Followback
PGSI: Problem Gambling Severity Index
NIDA-ASSIST: National Institute on Drug Abuse—Alcohol, Smoking and Substance Involvement Screening Test
NRT: Nicotine replacement therapy

## References

[1] Wood RT, Williams RJ. Internet gambling: prevalence, patterns, problems, and policy options. Final report prepared for the Ontario Problem Gambling Research Centre. Guelph: Ontario Problem Gambling Research Centre; 2009. https://opus.uleth.ca/bitstream/handle/10133/693/2009-InternetPPPP-OPGRC.pdf. Accessed 17 Mar 2020.

[2] Welte J, Barnes G, Tidwell M, Hoffman J, Wieczorek W (2015). Gambling and problem gambling in the United States: changes between 1999 and 2013. J Gambl Stud.

[3] Statistics Canada. 2014. http://www.statcan.gc.ca/tables-tableaux/sum-som/l01/cst01/health74beng.htm. Accessed 17 Mar 2020.

[4] Substance Abuse and Mental Health Services Administration. Results from the 2009 National Survey on Drug Use and Health: Volume I. Rockville: Summary of National Findings; 2010.

[5] McGrath DS, Barrett SP (2009). The comorbidity of tobacco smoking and gambling. A review of the literature. Drug Alcohol Rev.

[6] Grant BF, Hasin DS, Chou SP, Stinson FS, Dawson DA (2004). Nicotine dependence and psychiatric disorders in the United States: results from the national epidemiologic survey on alcohol and related conditions. Arch Gen Psychiatry.

[7] Smart RG, Ferris J (1996). Alcohol, drugs and gambling in the Ontario adult population, 1994. Can J Psychiatry.

[8] McGrath DS, Barrett SP, Stewart SH, McGrath PR (2012). A comparison of gambling behavior, problem gambling indices, and reasons for gambling among smokers and nonsmokers who gamble: evidence from a provincial gambling prevalence study. Nicotine Tobacco Res.

[9] Grant JE, Desai RA, Potenza MN (2009). Relationship of nicotine dependence, subsyndromal and pathological gambling, and other psychiatric disorders: data from the National Epidemiologic Survey on Alcohol and Related Conditions. J Clin Psychiatry.

[10] Lorains FK, Cowlishaw S, Thomas SA (2011). Prevalence of comorbid disorders in problem and pathological gambling: systematic review and meta-analysis of population surveys. Addiction..

[11] Dowling NA, Cowlishaw S, Jackson AC, Merkouris SS, Francis KL, Christensen DR (2015). Prevalence of psychiatric co-morbidity in treatment-seeking problem gamblers: a systematic review and meta-analysis. Aust N Z J Psychiatry.

[12] World Health Organization. Global status report on alcohol and health-2014: World Health Organization; 2014. https://apps.who.int/iris/bitstream/handle/10665/112736/9789240692763_eng.pdf. Accessed 17 Mar 2020.

[13] Grant JE, Kim S, Odlaug BL, Potenza MN (2008). Daily tobacco smoking in treatment-seeking pathological gamblers: clinical correlates and co-occurring psychiatric disorders. J Addict Med.

[14] Grant JE, Potenza MN (2005). Tobacco use and pathological gambling. Ann Clin Psychiatry.

[15] Petry NM, Oncken C (2002). Cigarette smoking is associated with increased severity of gambling problems in treatment-seeking gamblers. Addiction..

[16] Potenza MN, Steinberg MA, Mclaughlin SD, Wu R, Rounsaville BJ, Krishnan-Sarin S (2004). Characteristics of tobacco-smoking problem gamblers calling a gambling helpline. Am J Addict.

[17] Gooding P, Tarrier N (2009). A systematic review and meta-analysis of cognitive-behavioural interventions to reduce problem gambling: hedging our bets?. Behav Res Ther.

[18] Cowlishaw S, Merkouris S, Dowling N, Anderson C, Jackson A, Thomas S. Psychological therapies for pathological and problem gambling. Cochrane Database Syst Rev. 2012;11:CD008937. 10.1002/14651858.CD008937.pub2 PMC11955261 23152266

[19] Yakovenko I, Quigley L, Hemmelgarn BR, Hodgins DC, Ronksley P (2015). The efficacy of motivational interviewing for disordered gambling: systematic review and meta-analysis. Addict Behav.

[20] Hettema JE, Hendricks PS (2010). Motivational interviewing for smoking cessation: a meta-analytic review. J Consult Clin Psychol.

[21] Perkins KA, Conklin CA, Levine MD (2008). Cognitive-behavioral therapy for smoking cessation: a practical guidebook to the most effective treatments.

[22] Health M (2015). Annual statistics 2014–2015.

[23] Hodgins DC, El-Guebaly N (2000). Natural and treatment-assisted recovery from gambling problems: a comparison of resolved and active gamblers. Addiction..

[24] van der Maas M, Shi J, Elton-Marshall T, Hodgins DC, Sanchez S, Lobo DSS, Hagopian S, Turner NE (2019). Internet-based interventions for problem gambling: scoping review. JMIR Ment Health.

[25] Di Chiara G, Imperato A (1988). Drugs abused by humans preferentially increase synaptic dopamine concentrations in the mesolimbic system of freely moving rats. Proc Natl Acad Sci.

[26] Pontieri FE, Tanda G, Orzi F, Di Chiara G (1996). Effects of nicotine on the nucleus accumbens and similarity to those of addictive drugs. Nature..

[27] Zack M, Poulos CX (2004). Amphetamine primes motivation to gamble and gambling-related semantic networks in problem gamblers. Neuropsychopharmacology..

[28] McGrath DS, Barrett SP, Stewart SH, Schmid EA (2012). The effects of acute doses of nicotine on video lottery terminal gambling in daily smokers. Psychopharmacology..

[29] Barrett SP, Tichauer M, Leyton M, Pihl RO (2006). Nicotine increases alcohol self-administration in non-dependent male smokers. Drug Alcohol Depend.

[30] Reid MS, Mickalian JD, Delucchi KL, Hall SM, Berger SP (1998). An acute dose of nicotine enhances cue-induced cocaine craving. Drug Alcohol Depend.

[31] Spiga R, Schmitz J, Day J (1998). Effects of nicotine on methadone self-administration in humans. Drug Alcohol Depend.

[32] Barrett SP, Boileau I, Okker J, Pihl RO, Dagher A (2004). The hedonic response to cigarette smoking is proportional to dopamine release in the human striatum as measured by positron emission tomography and [11C]raclopride. Synapse..

[33] Zald DH, Boileau I, El-Dearedy W (2004). Dopamine transmission in the human striatum during monetary reward tasks. J Neurosci.

[34] Businelle MS, Kendzor DE, Rash CJ, Patterson SM, Coffey SF, Copeland AL (2009). Heavy smokers perform more poorly than nonsmokers on a simulated task of gambling. Subst Use Misuse.

[35] Bickel WK, Odum AL, Madden GJ (1999). Impulsivity and cigarette smoking: delay discounting in current, never, and ex-smokers. Psychopharmacology..

[36] Mooney ME, Odlang BL, Kim SW, Grant JE (2011). Cigarette smoking status in pathological gamblers: association with impulsivity and cognitive flexibility. Drug Alcohol Depend.

[37] Wulfert E, Harris K, Broussard J (2016). The role of cross-cue reactivity in coexisting smoking and gambling habits. Journal of Gambling Issues.

[38] Hodgins DC, Currie SR, el-Guebaly N (2001). Motivational enhancement and self-help treatments for problem gambling. J Consult Clin Psychol.

[39] Casey LM, Oei TPS, Raylu N, Horrigan K, Day J, Ireland M, Clough BA (2017). Internet-based delivery of cognitive behaviour therapy compared to monitoring, feedback and support for problem gambling: a randomised controlled trial. J Gambl Stud.

[40] Tolchard B, Battersby M (2013). Treatment completion in a cognitive behaviour therapy service for problem gamblers: clinical outcome study. J Addiction Res Ther.

[41] Melville KM, Casey LM, Kavanagh DJ (2007). Psychological treatment dropout among pathological gamblers. Clin Psychol Rev.

[42] Petry NM, Ammerman Y, Bohl J, Doersch A, Gay H, Kadden R, Molina C, Steinberg K (2006). Cognitive-behavioural therapy for pathological gamblers. J Consult Clin Psychol.

[43] Goslar M, Leibetseder M, Muench HM, Hofmann SG, Laireiter AR (2017). Efficacy of face-to-face versus self-guided treatments for disordered gambling: a meta-analysis. J Behav Addict.

[44] Toneatto T, Brennan J (2002). Pathological gambling in treatment-seeking substance abusers. Addict Behav.

[45] Simpson HB, Maher MJ, Wang Y, Bao Y, Foa EB, Franklin M (2011). Patient adherence predicts outcome from cognitive behavioural therapy in obsessive-compulsive disorder. J Consult Clin Psychol.

[46] Koçak ND, Eren A, Boğa S, Aktürk Ü, Öztürk Ü, Arınç S, Şengül A (2015). Relapse rates and factors related to relapse in a 1-year follow-up of subjects participating in a smoking cessation program. Respir Care.

[47] Smith DP, Battersby MW, Pols RG, Harvey PW, Oakes JE, Baigent MF (2015). Predictors of relapse in problem gambling: a prospective cohort study. J Gambl Stud.

[48] Merkouris SS, Thomas SA, Browning CJ, Dowling NA (2016). Predictors of outcomes of psychological treatments for disordered gambling: a systematic review. Clin Psychol Rev.

[49] Barrett SP, Darredeau C, Pihl RO (2006). Patterns of simultaneous polysubstance use in drug using university students. Hum Psychopharmacol.

[50] Dutra L (2008). A meta-analytic review of psychological interventions for substance use disorders. Am J Psychiatr.

[51] Ladd GT, Petry NM (2003). A comparison of pathological gamblers with and without substance abuse treatment histories. Exp Clin Psychopharmacol.

[52] Mueser KT, Noordsy DL, Drake RE, Fox L (2003). Integrated treatment for dual disorders: a guide to effective practice.

[53] Goudriaan AE, Oosterlaan J, De Beurs E, Brink WVD (2006). Neurocognitive functions in pathological gambling: a comparison with alcohol dependence, Tourette syndrome, and normal controls. Addiction..

[54] Bickel WK, Mueller TE (2009). Toward the study of trans-disease processes: a novel approach with special reference to a study of co-morbidity. J Dual Diagnosis.

[55] Abrams DB, Niaura R, Brown RA, Emmons KM, Goldstein MG, Monti PM (2003). The tobacco dependence treatment handbook: a guide to best practices.

[56] Cunningham JA, Hodgins DC, Keough M, Hendershot CS, Schell C, Godinho A (2020). Online interventions for problem gamblers with and without co-occurring unhealthy alcohol use: randomized controlled trial. Internet Interv.

[57] Cunningham JA, Hodgins DC, Mackenzie CS, Godinho A, Schell C, Kushnir V, Hendershot CS (2019). Randomized controlled trial of an Internet intervention for problem gambling provided with or without access to an Internet intervention for co-occurring mental health distress. Internet Interv.

[58] Manitoba Health, Seniors, and Active Living: Information management and analytics. Annual Statistics 2017-2018. Retrieved from https://www.gov.mb.ca/health/annstats/as1718.pdf. Accessed 17 Mar 2020.

[59] Center for Behavioral Health Statistics and Quality. 2018 Surveillance report of drug-related risks and outcomes. https://www.cdc.gov/drugoverdose/pdf/pubs/2018-cdc-drug-surveillance-report.pdf. Accessed March 17, 2020.

[60] Gainsbury S, Blaszczynski A (2011). Online self-guided interventions for the treatment of problem gambling. Int Gambl Stud.

[61] Cunningham JA (2016). Online interventions for problem gamblers with and without co-occurring mental health symptoms: protocol for a randomized controlled trial. BMC Public Health.

[62] Haug S, Schaub MP, Venzin V, Meyer C, John U (2013). Efficacy of a text message-based smoking cessation intervention for young people: a cluster randomized controlled trial. J Med Internet Res.

[63] Wiebe J, Mun P, Kaufman N (2006). Gambling and problem gambling in Ontario.

[64] Mark ME, Lesieur HR (1992). A feminist critique of problem gambling research. Br J Addict.

[65] American Psychiatric Association (2013). Diagnostic and statistical manual of mental disorders (5th ed.).

[66] Dube P, Kurt K, Bair MJ, Theobald D, Williams LS (2010). The P4 screener: evaluation of a brief measure for assessing potential suicide risk in 2 randomized effectiveness trials of primary care and oncology patients. Prim Care Companion J Clin Psychiatry.

[67] Saunders JB, Aasland OG, Babor TF, de la Fuente JR, Grant M (1993). Development of the alcohol use disorders identification test (AUDIT): WHO collaborative project on early detection of persons with harmful alcohol consumption—II. Addiction..

[68] Skinner HA (1982). The drug abuse screening test. Addict Behav.

[69] Tiet QQ, Ilgen MA, Byrnes HF, Harris AHS, Finney JW (2007). Treatment setting and baseline substance use severity interact to predict patients’ outcomes. Addiction..

[70] Hodgins DC, Currie SR, Currie G, Fick GH (2009). Randomized trial of brief motivational treatments for pathological gamblers: more is not necessarily better. J Consult Clin Psychol.

[71] Sobell LC, Sobell M (1992). Timeline followback: a technique for assessing self-reported ethanol consumption. Measuring alcohol consumption: Psychosocial and biological methods.

[72] Weinstock J, Whelan JP, Meyers AW (2004). Behavioral assessment of gambling: an application of the timeline followback method. Psychol Assess.

[73] Rueger SY, Trela CJ, Palmeri M, King AC (2012). Self-administered web-based timeline followback procedure for drinking and smoking behaviours in young adults. J Stud Drugs Alcohol.

[74] Kim S-K, Grant JE, Potenza MN, Blanco C, Hollander E (2009). The Gambling Symptom Assessment Scale (G-SAS): a reliability and validity study. Psychiatry Res.

[75] Ferris J, Wynne H. The Canadian problem gambling index: final report. Submitted for the Canadian Centre on Substance Abuse; 2001.

[76] Heatherton TF, Kozlowski LT, Frecker RC, Fagerström K (1991). The Fagerstrom test for nicotine dependence: a revision of the Fagerstrom tolerance questionnaire. Br J Addict.

[77] Rohsenow DJ, Martin RA, Tidey JW, Monti PM, Colby SM (2013). Comparison of the Cigarette Dependence Scale with four other measures of nicotine involvement: correlations with smoking history and smoking treatment outcome in smokers with substance use disorders. Addict Behav.

[78] Hughes JR, Hatsukami D (1986). Signs and symptoms of tobacco withdrawal. Arch Gen Psychiatry.

[79] National Institute on Drug Abuse (2009). NIDA-modified ASSISTprescreen VI.0. https://www.drugabuse.gov/sites/default/files/pdf/nmassist.pdf. Accessed 1 Sept 2017.

[80] Hodgins DC, Fick GH, Murray R, Cunningham JA (2013). Internet-based interventions for disordered gamblers: study protocol for a randomized controlled trials of online self-directed cognitive behavioural motivational therapy. BMC Public Health.

[81] Fiore MC, Jaén CR, Baker TB (2008). Treating tobacco use and dependence: 2008 update. Clinical Practice Guideline.

[82] Murray E, White IR, Varagunam M, Godfrey C, Khadjesari Z, McCambridge J (2013). Attrition revisited: adherence and retention in a Web-based alcohol trial. J Med Internet Res.

[83] Mohr DC, Cuijpers P, Lehman K (2011). Supportive accountability: a model for providing human support to enhance adherence to eHealth interventions. J Med Internet Res.

[84] Brorson HH, Arnevik EA, Rand-Hendriksen K, Duckert F (2013). Drop-out from addiction treatment: a systematic review of risk factors. Clin Psychol Rev.

[85] Keough MT, O'Connor RM, Colder CR (2016). Testing the implicit and explicit cognitions underlying behavioral inhibition system-related drinking in young adults. Alcohol Clin Exp Res.

[86] Karlsson A, Hakansson A (2018). Gambling disorder, increased mortality, suicidality, and associated comorbidity: a longitudinal nationwide register study. J Behav Addict.

