# Supplementary material for: Developing and testing the effectiveness of a novel online integrated treatment for problem gambling and tobacco smoking: a protocol for an open-label randomized controlled trial
Source: Trials. 2020 Nov 19;21:937. doi: 10.1186/s13063-020-04867-1 (PMC7678080; doi:10.1186/s13063-020-04867-1)
Supplement: Supplementary file 1 — Additional file 1. Informed Consent Form [file 13063_2020_4867_MOESM1_ESM.docx]

Appendix A: Consent Form

**Informed Consent Form**

**Study Name**: Developing and testing the effectiveness of a novel online integrated treatment for problem gambling and tobacco smoking: A randomized controlled trial

**Researchers**: Dr. Matthew Keough, Department of Psychology, 4700 Keele St, York University, North York ON, M3J 1P3; keoughmt@yorku.ca

**Purpose of the Research:** This research is being conducted to study the effectiveness of an online, self-help integrated treatment for problem gambling. You may also be asked to work on changing smoking behaviours, depending on which condition you are in. You are being asked to take part in this because you expressed interest to the advertised study.

Research has shown that problem gambling and tobacco smoking are highly comorbid. Problem gamblers who smoke tend to: have more severe gambling problems, experience stronger gambling urges, have other mental disorders, bet larger sums of money, spend more time in gambling activities, and have greater financial problems. Accordingly, it might be helpful to reduce your smoking as well, while you are trying to get a grip on your gambling.

Previous studies have treated gambling on its own or tobacco smoking on its own. To date, there has not been a treatment that has targeted treating both at the same time. Considering how often these conditions occur together, it is important to see if combining their treatment results in beneficial outcomes. In the current research, the researchers aim to design and test an online, self-help intervention for co-occurring problem gambling and tobacco smoking. It will draw on strategies from Cognitive Behavioural Therapy (CBT) and Motivational Interviewing (MI) – both which have been shown to be successful in treating problem gambling and tobacco smoking. These outcomes will be compared to a group that receives treatment for problem gambling only. The online format will provide benefits like easier accessibility and the ability to reach communities that are dispersed throughout the province. This research will provide insight into a potential new intervention for treating two highly co-occurring conditions. Part of the integrated treatment will be us advertising you to use nicotine replacement therapy (NRT) in the form of nicotine patch. If you are in the integrated treatment, we will provide vouchers so you can use nicotine patches during the course of treatment.

The researchers are hoping to recruit 300 participants for this study. Half (150) of these individuals will receive the integrated treatment, where both gambling and smoking behaviours will be addressed, and the other half (150) will receive the problem gambling treatment only.

**What You Will Be Asked to Do in the Research**: If you consent to participate in the research, you will need to register on the study website and verify your account. You can do this via a link you will be sent over e-mail. You will need to verify your account as well as create a username and password in order to access the treatment website. You will use this information for all log-ins throughout the study.

Once you have provided informed consent and are registered on the treatment website, you will be asked to complete a baseline assessment in order to determine whether you are eligible to participate. Eligible participants will be those that: are aged 19 years old+, have at least moderate levels of gambling, report current daily smoking, are fluent in English, and have weekly internet access.

If you consent to participate in this research, you will be “randomized” into one of two study groups described below. “Randomized” means that you are put into a group by chance, like flipping a coin. The two groups are the study treatment group (treatment for both gambling and smoking) and the gambling only (i.e., control) condition. You will have a one in two chance of being placed in the study treatment group (integrated problem gambling and tobacco smoking) and a one in two chance of being placed in the gambling only group. Importantly, the participants assigned to the gambling only group will receive immediate access to an empirical, gambling only treatment. At the conclusion of the study, they will receive access to the integrated gambling and smoking treatment, including vouchers for NRT.

If you take part in this study, you go through the following steps:

1) After completing your consent form, you will complete a series of brief questionnaires. Following the completion of these forms and if deemed eligible, you will be randomly assigned a group. You will receive an active link to access the treatment, either the study treatment group or the gambling-only group.

2) You will need to create a username and password in order to access the treatment website. All the details will be provided for this.

3) After creating an account, study personnel will contact you by phone to ensure that your account is verified. Depending on which condition you are placed in, you may be asked to provide your mailing address in order to be mailed NRT for the duration of the program. In order to initiate your participation in this trial, we need to have a phone call with you.

4) Once the link is available, you will participate in the online, self-help 8-week treatment. At the end of the program, you will be asked to complete the same brief questionnaires. You will have 8-weeks to work through seven modules at your own pace. Throughout the modules, there will also be exercises that require additional practice. Overall, you should be prepared to commit approximately 1-3 hours/week.

5) Six months following the completion of the online program, you will be asked to complete the same series of brief questionnaires. We may contact you by phone to complete the follow-up questionnaires.

You can stop participating at any time. However, if you decide to stop participating in the study, we encourage you to talk to the study staff first. Although there are no serious consequences of a sudden withdrawal from the study, it is helpful for the study staff to understand why you are withdrawing or in what ways we could have made your participation more comfortable, as this may inform future studies.

It is important to know that this program is meant to be mainly self-guided – meaning that you work through the program at your own pace. However, you will be interacting occasionally with an intervention support person by email, who is an individual with training in clinical psychology. They will send you tips and feedback every so often, and will answer your questions as you go through the program. The function of the intervention support person is help you stay on course during the self-help program, and therefore, the intervention support person is not meant to be a therapist. If at any point you notice a sudden change in your wellbeing and feel you need more intensive support (e.g., in person counselling) please see the “Help Me” tab on the intervention website for resources in your area.

If you are interested in receiving the results of this study, please provide the research team with your contact information in the space provided at the end of this form. You will receive an email summary of the study once it has been completed. Please provide your email in the space provided at the end of this form if you are interested.

As a token of appreciation for your participation in the study, you will be given a $20 Amazon.ca gift card per assessment. A bonus of $20 for completing all three assessments, and $20 for completing at least five of the treatment modules (max compensation per participant = $100).

**Risks and Discomforts**: Although there are no known risks of participating in a combined CBT and MI intervention, it is possible that you may find some of the content during treatment difficult. Some individuals who have difficulty with emotions sometimes get thoughts of wanting to harm themselves. If you report significant thoughts of harming yourself and/or plans at the beginning, you will be given a recommendation to visit your local doctor or hospital for support as soon as possible. You will also be provided with ongoing supports via the menu item “Help Me” on the intervention website. This will include a list of mental health services, including community resources, public and private psychologists, hospitals, and helplines. You will have access to these resources and may use them at any time. Dr. Matthew Keough, a clinical psychologist (supervised practice) will also be available to speak with you should serious issues arise and will make sure a safe course of action is followed. If you feel uncomfortable at any time during treatment, you are free to stop participating at any time.

There are some potential risks of using over-the-counter NRT nicotine patches. These can include skin irritation, headache, dizziness, etc. It is important to read the packaging of the patches and speak with the pharmacist if you have further questions.

If you become uncomfortable during this training, you are free to immediately stop participating.

**Benefits of the Research and Benefits to You**: There are also a number of potential benefits of the current study. First, you will be given skills to cope with both problem gambling and/or tobacco smoking. As a result, you may experience an improvement in your overall mental and physical health. We also hope that by offering the program online, we will be able to reach as many people as possible who otherwise may not have had access to treatment. We hope that the information in this study can be used to benefit other individuals struggling with problem gambling and smoking in the future. The results of this study may also be used by future researchers and professionals in order to continue improving the health and well-being of individuals struggling with mental health issues. There are also a number of benefits of using NRT nicotine patches, including reducing smoking and curbing cravings. Finally, the program is being offered free of change, when standard rates of therapy in the province are as high as $225/hour.

**Voluntary Participation and Withdrawal**: Your participation in the study is completely voluntary and you may choose to stop participating at any time. You have the right to withdraw at any time without penalty, financial or otherwise. Your decision not to volunteer, to stop participating, or to refuse to answer particular questions will not influence the nature of your relationship with York University either now, or in the future. Should you wish to withdraw after the study, you will have the option to also withdraw your data up until the analysis is complete.

**Confidentiality**: All information you supply during the research will be held in confidence and unless you specifically indicate your consent, your name will not appear in any report or publication of the research. Information gathered in this research study may be published or presented in public forums such as research conferences; however, any identifying information will not be used or revealed. All participants will be assigned a de-identifying (“subject ID”) so that all identifying information (e.g., email) can be removed from individual data sets, and the key linking subjects to their identifiers will be kept on a password-protected document kept separate from the data. However, despite our best efforts to keep your personal information confidential, absolute confidentiality cannot be guaranteed. All information obtained during the study will be held in strict confidence. You will be identified with a study number only. Your data will be safely stored in a password-protected document on a cloud-based server (e.g., Google drive) and only research team members will have access to this information. We will destroy the identifying information once data collection is completed and all NRT has been mailed. We will delete the online document where this information is housed. The de-identified data will be retained indefinitely so we can continue to analyze the data for future research. Confidentiality will be provided to the fullest extent possible by law.

The researcher(s) acknowledge that the host of the online survey (e.g., Qualtrics, Survey Monkey etc.) may automatically collect participant data without their knowledge (i.e., IP addresses.) Although this information may be provided or made accessible to the researchers, it will not be used or saved without participant’s consent on the researchers’ system. Further, “Because this project employs e-based collection techniques, data may be subject to access by third parties as a result of various security legislation now in place in many countries and thus the confidentiality and privacy of data cannot be guaranteed during web-based transmission.

**Questions About the Research?** If you have questions about the research in general or about your role in the study, please feel free to contact Dr. Keough either by telephone at 416-736-2100 ext. 33415 or by e-mail ([keoughmt@yorku.ca](mailto:keoughmt@yorku.ca)). This research has received ethics review and approval by the Human Participants Review Sub-Committee, York University’s Ethics Review Board and conforms to the standards of the Canadian Tri-Council Research Ethics guidelines. If you have any questions about this process, or about your rights as a participant in the study, please contact the Sr. Manager & Policy Advisor for the Office of Research Ethics, 5^th^ Floor, Kaneff Tower, York University (telephone 416-736-5914 or e-mail [ore@yorku.ca](mailto:ore@yorku.ca)).

**Legal Rights and Signatures**:

I consent to participate in “Developing and testing the effectiveness of a novel online integrated treatment for problem gambling and tobacco smoking: A randomized controlled trial” conducted by Dr. Keough. I have understood the nature of this project and wish to participate. I am not waiving any of my legal rights by signing this form. My signature below indicates my consent.

**Signature Date**

Participant

**Signature Date**

Principal Investigator
